# Three Thousand a Year:—A Soliloquy

**Published:** 1861-07

**Authors:** 


### Art. VII.—THREE THOUSAND A YEAR:—A SOLILOQUY.

—— Certainly I am "doing" about 3000*l.* a year. I think there can be no mistake about it. What makes me think so is, that for the last week or so I have been putting on my books at least 9*l.* a day. This is as good as I had any right to calculate upon. It was very prudent on my part in fixing on a house in this conspicuous situation. It is a duty to make oneself known, and to place oneself within easy access of the world. The best men have done so as a matter of course—such as Sir Astley Cooper, or Sir Henry Halford. My friend Eusebius and his partner, together, at the other end of London, do at least 7000*l.* a year. Let me see.—They have four carriages, seven horses, two grooms, and three assistants. As early as seven in the morning they start to see their out-of-town patients. Then they return in time for their home customers—I mean patients—about eleven or twelve o'clock, noon. Next, they lunch, and after that drive round to see their town patients. I know Eusebius declares that their run of business is often more like 9000*l.* than 7000*l.* a year; but we will make some allowances for a very pardonable exaggeration in this respect. It is so easy to deceive oneself, without meaning to do so, in the full tide of reputation and success. I know that Eusebius is called to the greatest distances. He refused a visit to Worcester the other day, and a second to Ramsgate, because he thought the fees too small. Well, I do not venture to suppose I shall rival him in prosperity. But certainly, things look very well just now, and I am on the fair way to make 3000*l.* a year, if I am not making it already. I am attending Lady ——'s lady's-maid, and one of the daughters. They are worth looking after, for their connexions are extensive, and I have already gained some introductions through their good word. Then, there is the rich banker in —— lane, and all his family. It is worth something to be known as in attendance on a house of that description. They pay well; and, in fact, are so rich that they have only to ring the bell and ask for whatever they want. A powdered footman, with a sweet-smelling note, calls for me almost every day. I have also Lord This upon my list, and his lordship has just introduced me to his stockbroker, so well known in the money market. I have no doubt I shall attend the Governor of the Bank of England next. This would be a grand catch. I gained great credit from the dexterity with which I rescued the Honourable Mrs. X. Y. Z.'s daughter from the imminent danger she was in through the neglect or mismanagement of her learned and distinguished medical adviser. The President of the Royal College of Physicians will never forgive my success in that quarter. As for the Honourable Mrs. X. herself, she was so fully convinced of my merit, that she actually made me a present of my own bust in marble, the size of life, from the studio of Chantrey, and insisted on my placing it in a visible part of my entrance-hall. She likewise requested the erudite Mr. Simon Chatterly to compose a Latin inscription to be inscribed in letters of gold around the base. The Greek Professor of ——, whom I am now attending, says it is the finest piece of Latinity he ever read. He has just done me the honour of requesting me to favour him with one of my photograph likenesses, which he will take the earliest opportunity of placing before a very distinguished personage, accompanied with an encomium on my professional talents. That was a capital turn of fortune in my favour—I mean my being called to that case beyond Oxford—a very good patient, and well worth looking after. It was a severe case, and it turned out well. The old fellow, I find, is a distant relation of the Marquis of Carabas, whose son is the M.P. for the county, and has his town residence close by mine in London. I must try and get in there—a good connexion. My neighbour, Tom Blank, attends the family. A good enough sort of man, but rather easy and antiquated—a by-gone. I must keep a sharp look-out, and seize the first opportunity of getting my foot in the doorway. And, then, that fellow Wayse,—why, Dr. Wayse, when he was here, did nothing—absolutely nothing. Now, since he has turned homœopath he does 1000*l.* a year, and without the smallest trouble. "Why, Wayse," I said to him the other day, when we met at the Eastern Counties' station, —"Why, Wayse, they tell me you are making your fortune—is that the case? Wish you joy of it, my good fellow, with all my heart." "O yes," says Wayse, "it is quite true." "And you are also a homœopath," I continued; "and is that true also?" "No, not exactly a homœopath," returns Wayse. "Not?—What are you then?" "An eclectic," says Wayse, rather demurely. "An eclectic. What is that?" "Why," says Wayse, "I mean I am an eclectic in the real meaning of the word, for I choose what is best out of everything, whether homœopathy or allopathy. My object is the good of my patient." "Capital! I understand: very sensible, and exactly to the point." After parting, I remembered that Wayse had lately published a pamphlet, in which, among other things, he maintains that a few drops of laudanum in a tumbler of water is an infallible cure for apoplexy. A *few* drops did I say? It was a single drop, if not less than that. How very curious! I wonder if Dr. Wayse believes what he says? Let me see! Apoplexy—what is the cause of apoplexy? The causes are numerous. First of all, simple congestion. Perhaps he means that, because he states, that as a full dose of laudanum produces apoplectic congestion, so an infinitesimal dose of the same drug will cure it. I know he said so; I heard him say it; and I have read his words to this effect in print. Well, this is certainly something quite new. Then, the next cause is, What? Let us say an atheromatous condition of the arteries. Will a few drops of landanum cure this? Atheromatous arteries—I do not quite like that word—it is not simple enough. The other day, when I was in consultation with Dr. F., I said I thought the arteries in our patient's head were atheromatous. "What do you mean by atheromatous?" asked Dr. F., his eyes twinkling, and glancing at me rather slily I fancied. "Atheromatous—" I said, meaning to explain myself, but my memory was so treacherous at the moment, that I could not recollect the primitive derivation of the word, although I remembered it was Greek. Just as I turned to explain my meaning, I caught Dr. F.'s eye steadily examining my carriage and pair, drawn up alongside of his, as we were both looking out of the dining-room window into the street. "What do you think of my two greys, Dr. F.?" I inquired, breaking in upon his apparent abstraction; "they are capital carriage horses, and I gave 60*l.* a piece for them—they do their work excellently." "60*l.* a piece!—you must be a rich man," said Dr. F., politely taking leave of me, and smiling as he turned away. A distinguished man that Dr. F., thought I to myself, and evidently accustomed to high people. I must do my best to make up to him; it's worth my while to stand well in his good graces. Bother that word *atheromatous*; why did I use it? I ought to have said *degeneration*, which would have done just as well, and sounded quite as pathological. I do not like pointed questions—they argue a malevolent disposition. Why, the other day, as I was talking to that old-fashioned fellow H., in the street, and telling him of my doing 3000*l.* a year, he said bluntly in return, "Then you pay income-tax on 3000*l.*" "No, not exactly so," I replied. "How so?" retorted H.; "how do you manage to get off?" "Why," I replied, "if you must know, I have not made up my books yet, so I cannot precisely say so." "Then, in fact," continued H., in the coolest tone possible, "you do not know for certain that you are making 3000*l.* a year, but only imagine it." Nothing could have been in worse taste. I wished him good bye as quickly as possible. But this is not all. One day I told him I had a case of fever under my care of great severity, arising from poisoned blood. "Poisoned blood," he said eagerly; "how do you know it was poisoned blood?" He is the most provoking creature in the world; merely a reading man, who knows nothing of practice. For my part, I never read. I am a practical man. I read nature at the bedside. Of course it was poisoned blood—no one could doubt it, and I said so to him. "Yes," he replied, "you have no doubt of it, I see. But, now, tell me. If you had two cups of blood, freshly drawn, before you, one from a healthy person, and the other from a blood-poisoned patient, could you tell me the difference simply by looking at them, or have you any chemical or microscopical proof, by which you can distinguish the one from the other—the poisoned from the healthy blood? If so, pray tell me, for I am desirous of collecting all the information I can on this topic." Now, was there ever anything half so tiresome as this fellow? But this is the way with these reading men, they are always for pushing their inquiries to the utmost, and are content with nothing but absolute proof and certainty. Just as if I could tell him anything of the sort, or had time to make such minute inquiries. No, not I; I have something better to look after. So I cut the matter short by wishing him good day, stepped into my carriage, and drove off. There is no escaping, however, from folks of this kind. Not long after, I met him again, just after that distressing case of suicide, which made so much noise at the time, and in which I was summoned to appear as the medical witness at the inquest. "Why," exclaimed H., "what a remarkable case that was—the man spoke after his throat was cut! I should never have given credit to anything so remarkable, except upon your testimony; and I have entered it in my note book, under your name thus: A man cut his throat through the bronchial tubes, and yet kept crying out, 'Oh! doctor, save me,' and lived three weeks afterwards. I think it so very remarkable, that I shall keep it by me to publish on some future occasion, upon the authority of your good name. And it is the more remarkable," he continued, for it was impossible to stop him, "because the cut went through, not the larynx, which is above the sternum, but through the two bronchi, or bronchial tubes, which are below and behind the sternum, and the cut missed wounding the chief arteries which lie in front of, or contiguous to, the bronchi, and must in ordinary cases have been first cut through before the bronchi could be reached. Most remarkable! I have made a note of it, and will publish it whenever I am required to place upon record *The Curiosities of Surgery.*"* This was too bad. I saw I could no longer keep terms with him. So I took the advice of my patient and friend, Major Longbeard, and resolved to disembarrass myself of a troublesome acquaintance of this kind at the first convenient opportunity. I had not long to wait. One day, as I was driving up to a patient's door in my open carriage, H., who was passing by at the time, stepped forward with alacrity to help me to alight. I touched his hand with the tips of my fingers, treated him with the disdain he deserved, and cut him dead on the spot.

---

### Art. VIII.–THE STATE OF LUNACY IN SCOTLAND.

* *Extract from the printed report of the Inquest* (Daily papers.) "By the foreman: I never once saw a symptom of insanity in him. Another juror: If you say his mind was not at all impaired, how do you account for this act? Witness: Temporary insanity. You and I are liable to temporary insanity. I once was called in to attend a man who had cut his throat; but he had only cut through the bronchial tubes, and had not divided the carotid arteries; and he kept crying out, 'Oh! doctor, save me, save me.' I attended to him, and then had him removed to a neighbouring hospital, and he lived for three weeks." The jury were perfectly satisfied with the evidence, and what was still more surprising, so was the coroner.

